# Moving forward in carcinogenicity assessment: Report of an EURL ECVAM/ESTIV workshop

**DOI:** 10.1016/j.tiv.2017.09.010

**Published:** 2017-12

**Authors:** Raffaella Corvi, Federica Madia, Kathryn Z. Guyton, Peter Kasper, Ruthann Rudel, Annamaria Colacci, Jos Kleinjans, Paul Jennings

**Affiliations:** a European Commission, Joint Research Centre (JRC), EU Reference Laboratory for Alternatives to Animal Testing (EURL ECVAM), Ispra, (VA), Italy; b Monographs Programme, International Agency for Research on Cancer, Lyon, France; c Federal Institute for Drugs and Medical Devices (BfArM), Bonn, Germany; d Silent Spring Institute, Newton, United States; e Centre for Environmental Toxicology and Risk Assessment, Environmental Protection and Health Prevention Agency, Emilia Romagna Region, Italy; f Department of Toxicogenomics, Maastricht University, Maastricht, The Netherlands; g Division of Molecular and Computational Toxicology, Amsterdam Institute for Molecules, Medicines and Systems, Vrije Universiteit Amsterdam, HZ Amsterdam, The Netherlands

**Keywords:** Carcinogenicity, Alternative methods, Rodent bioassay, Toxicogenomics, Mechanisms, Cancer hallmarks, CTA

## Abstract

There is an increased need to develop novel alternative approaches to the two-year rodent bioassay for the carcinogenicity assessment of substances where the rodent bioassay is still a basic requirement, as well as for those substances where animal use is banned or limited or where information gaps are identified within legislation. The current progress in this area was addressed in a EURL ECVAM- ESTIV workshop held in October 2016, in Juan les Pins. A number of initiatives were presented and discussed, including data-driven, technology-driven and pathway-driven approaches. Despite a seemingly diverse range of strategic developments, commonalities are emerging. For example, providing insight into carcinogenicity mechanisms is becoming an increasingly appreciated aspect of hazard assessment and is suggested to be the best strategy to drive new developments. Thus, now more than ever, there is a need to combine and focus efforts towards the integration of available information between sectors. Such cross-sectorial harmonisation will aid in building confidence in new approach methods leading to increased implementation and thus a decreased necessity for the two-year rodent bioassay.

## Highlights

• Need to develop novel alternative approaches to the two-year rodent cancer bioassay
• Insight into carcinogenicity mechanisms is essential to drive new developments.
• Direct efforts towards the integration of all available information, also across endpoints.
• Cross-sector harmonisation to build confidence in new approach methods

## 1 Introduction

The approaches for evaluating the carcinogenic potential of substances, including prioritizing and selecting agents for rodent carcinogenicity studies, differ substantially across sectors. Nonetheless, the two-year rodent bioassay has remained the “gold standard” for carcinogenicity testing for nearly half a century. As from the first OECD Test Guideline release in 1981, the design has remained almost unaltered. Human carcinogens, when tested adequately, have all tested positive for carcinogenicity in one or more animal species (Tomatis et al., 1989, Wilbourn et al., 1986). However, several issues concerning the application of rodent bioassay data to predicting human cancer risks have emerged, with notable challenges in both anticipating the potential human cancer target organs and in quantitative risk estimation, confounded by differences in criteria for interpreting positive findings (Contrera et al., 1997, Knight et al., 2006, Paparella, 2016, Paules et al., 2011, Rudel et al., 2007). For instance, the reproducibility of positive findings in animals (*i*.*e*., increased tumours in more than one sex, species or bioassay) is an important consideration in IARC Monograph evaluations (International Agency for Research on Cancer, 2006), which also entail integration with human cancer and mechanistic findings. Moreover, the rodent bioassay, as originally designed, does not take into account windows of susceptibility over the life-time, and so may not have adequate sensitivity to detect agents, such as endocrine active chemicals, that alter susceptibility to tumours (Birnbaum and Fenton, 2003).

The need for clear evaluation guidelines is underscored by the assertion that rodent-specific mechanisms of carcinogenicity, differences in safety margin of exposures, and/or differences in metabolism, confound interpretation of rodent carcinogenicity studies of pharmaceuticals (Friedrich and Olejniczak, 2011, Sistare et al., 2011). Furthermore, these studies are extremely time and resource-consuming and the high animal burden has raised ethical concerns. Hence, there is a strong demand for rational prioritization schemes as well as for alternative non-animal assessment strategies and methods in the area of carcinogenicity (Annys et al., 2014, Creton et al., 2012, Jacobs and Brown, 2013, Jacobs et al., 2016). Yet, the recourse to currently available alternatives, has been very limited and is quite variable across sectors. Among the main bottlenecks are (i) the difficulties in defining how to meaningfully apply individual *in vitro* tests in the context of other available information, (ii) lack of a complete mechanistic understanding underlying carcinogenicity and (iii) taking the previous two points into consideration what is the regulatory implication of a positive or negative carcinogenicity test result in an *in vitro* assay to the fate of the chemical.

Here we present the outcome of the EURL ECVAM/ESTIV workshop on "the way forward in carcinogenicity assessment" which took place in October 2016 in conjunction with the ESTIV congress in Juan Les Pins.

## 2 Regulatory background

Regulatory strategies for testing carcinogenicity have diversified significantly across legislations depending on the type of substance, while maintaining two key elements: the testing for genotoxicity *in vitro/in vivo* and the two-year rodent bioassay. For industrial chemicals, requirements are based on a tiered-testing approach and on the annual amount of substance produced, to which potential exposure and degrees of exposure are linked. Carcinogenicity testing is required only for the high tonnage level and mainly for mutagens category 3 (GHS category 3). For all the new plant protection products and non-genotoxic new active biocides, the testing of carcinogenicity is required in two different species. Exposure to these products and their breakdown products is of major concern in occupational settings. On the other hand, for the general population which is exposed to very low doses for long periods of time, the concern is related to persistent metabolites and residues. Carcinogenicity of metabolites and residues is evaluated on a case-by-case basis. Of high concern are also residues of veterinary drugs in food for human consumption. It is a priority of this sector to rely on genotoxicity testing and structural similarities, so that positive results from those studies are further tested. Only when results from genotoxicity tests are clearly negative, no structure alerts are identified and human exposure is negligible, can animal testing be waived. Human medicines are commonly administered at high doses to reach the effective pharmacological dose, with short or chronic exposures. Carcinogenicity testing is performed mainly for drugs for which a chronic administration is foreseen. In this case, a test-battery approach is used starting always with genotoxicity *in vitro* testing followed by *in vivo* testing of genotoxicity and carcinogenicity. In contrast, no *in vivo* testing is allowed since March 2013 for cosmetic ingredients and carcinogenicity is predicted on the basis of alternative approaches only, relying mainly on *in vitro* genotoxicity testing (EC Regulation 1107, 2009, EC Regulation 1223, 2009, EC Regulation 1272, 2008, EC Regulation 1907., 2006, EU Regulation 283, 2013, EU Regulation 528, 2012, ICH S1, 2012, ICH S1A., 1996, SCCS, 2015, VICH GL28, 2005). This limited assessment for carcinogenicity potentially increases the probability of consumers being exposed to cosmetics ingredients which may promote tumours, alter hormone responsiveness of tissue, or influence cancer risk through other non-genotoxic mechanisms.

These cross-sectorial differences in regulation are mostly due to the extent of human health risk for each product use, the level of exposure to humans and the environment, the type of new products developed, economic issues and animal-welfare concerns.

## 3 Analysis of carcinogenicity testing for regulatory purposes in the EU

The European Union Reference Laboratory for Alternatives to Animal Testing (EURL ECVAM) has carried out an analysis of carcinogenicity testing requirements and assessment approaches across different sectors within the European Union (Madia et al., 2016). This consisted of a systematic review of the different testing requirements and the number of animals used *per se*ctor, an estimation of the number of carcinogenicity and genotoxicity studies conducted or waived in respect of the number of substances authorized per sector per year and a review of the type of justifications for waiving the two-year bioassay.

Three Rs initiatives have promoted several changes within regulatory toxicology testing since their first legal embedment in the 1986 EU Directive on the use of laboratory animals. Though, according to the latest figures, there has been a minimal decrease in the animal testing burden used for cancer studies (at least until 2011). In terms of absolute numbers this reduction could be regarded as negligible, as assessment of carcinogenicity *per se* is utilising fewer animals overall in comparison with other regulatory toxicity areas (*e*.*g*. acute, repro-, chronic toxicity, *etc*.), representing 1% of all toxicity testing (DG ENV Report, 2013). However, in terms of animal welfare, a single cancer study involves a large number of rodents, induces extended suffering, implies a long-lasting period of data analysis and is extremely resource-consuming (Adler et al., 2011). A significant number of carcinogenicity studies are performed in the area of basic research, mainly within academia (DG ENV Report, 2013), though they are usually not referred as to the standard two-year bioassay used for toxicological regulatory purposes.

The two-year bioassay is frequently conducted within the plant protection products sector [> 60%], even if a decrease of the number of substances tested likewise has been observed between years 2011 and 2014 (Madia et al., 2016). The majority of new active substances (10 per year, on average) are tested in a two-year cancer bioassay study or a combined chronic/carcinogenicity rat study, often in combination with a second study in a second rodent species, even though the relevance of the latter has been questioned (Annys et al., 2014, Billinton et al., 2010, Van Oosterhout et al., 1997). The Plant Protection Products Regulation (EC Regulation 1107, 2009) foresees the use of alternative models instead of the second species if scientifically justified, however this is rarely implemented. The carcinogenicity study is waived mainly on the basis of expected limited general population exposure risk, when it is technically not feasible, as in the case of some natural products or microorganisms or on the basis of lack of genotoxicity effect of the substance. In this regard, a conspicuous amount of substances are tested in *in vivo* genotoxicity studies.

The use of alternative approaches has been observed more frequently in the biocide sector which accounts for 12 authorizations per year approximately, 2–5 referring to new substances. The use of read-across data has been reported in several authorization dossiers for either the testing of carcinogenic or genotoxic potential. Opportunities for waiving carcinogenicity testing of biocidal products are similar to those described above for plant protection products. Overall, the two-year bioassay has been performed on 30% of biocidal substances (Madia et al., 2016).

Within the pharmaceuticals sector, a substantial portion of authorized human medicines (on average 35 new substances per year) undergo carcinogenicity testing (as observed either in 2011 or in 2014) and the use of alternative approaches is rarely considered. Within this sector, the two-year bioassay is not conducted for specific classes of therapeutic/diagnostic agents when it is not scientifically relevant or technically feasible. The introduction of specific shorter-term carcinogenicity studies, as the transgenic mouse model, seemed at first to impact positively on the 3Rs, showing promises for more technical specificity and impact on animal number. However, the transgenic model has resulted not to be a real reduction model because of the amount of animals needed for the breeding of the specific knockouts (Daneshian et al., 2015, Ormandy et al., 2011).

In the case of veterinary medicines (on average 10 new substances per year), the percent of authorized substances tested for carcinogenicity were approximately 24% in 2011 and none of the products authorized in 2014 have been tested for this endpoint, because they were mostly vaccines and biotechnology-derived proteins (Madia et al., 2016).

Since 2009 following the full introduction of REACH Regulation (EC Regulation 1907., 2006) up to the date of this analysis (Madia et al., 2016), only two industrial chemicals (> 1000 tons/year) have been tested for carcinogenicity, following acceptance of the submitter's new testing proposals by the European Chemical Agency (ECHA). Carcinogenicity studies are not expected to be conducted for the 2018 deadline registration due to reduced testing requirements for lower tonnage band chemicals. Though, new phase-in substances may be registered for which carcinogenicity testing should be considered. The impact of carcinogenicity testing on the authorization of substances has been null within the cosmetics sector. Yet, no cancer study has been performed since 2003, ten years ahead the full ban of *in vivo* testing of March 2013.

The analysis reported above has shown differences, not only in testing approaches across sectors and in the demand of carcinogenicity studies, but also in the opportunities for waiving the two-year study for certain types of substances. Waivers are often supported by the absence of structural alerts and lack of genotoxic potential and/or specific properties. Though, risk-based information regarding destination of use, target population and foreseen exposure levels are also considered and these vary substantially across sectors. Moreover, different regulations with specific testing requirements and levels of concerns are in place. For instance, the EU plant protection products law forbids the marketing of active substances which can cause cancer, while for certain types of pharmaceuticals instead, an intrinsic carcinogenic potential may be tolerated (Madia et al., 2016). At present, we are not in a position to apply similar waiving conditions across sectors. A substantial portion of new substances, such as bio-pesticides and new drugs (biotechnology-derived ones) currently entering the global market, are characterized by specific features that make their testing not technically feasible with the current available methods. Consequently, their safety characterization is rather difficult raising regulatory and technical challenges that call for new tools.

## 4 Alternative approaches to rodent long-term studies for carcinogenicity assessment of pharmaceuticals

According to International Council for Harmonisation (ICH) guidance documents carcinogenicity studies need to be conducted to support marketing application for most small molecule pharmaceuticals (ICH S1A., 1996, ICH S1B, 1997).

In August 2013 an ICH Regulatory Notice Document (RND) has been posted by the Drug Regulatory Authorities (DRAs) in the ICH regions (US Food and Drug Administration, European Medicines Agency, Pharmaceuticals and Medical Devices Agency in Japan, Health Canada, and SwissMedic) announcing the evaluation of an alternative approach to the 2-year rat carcinogenicity test (ICH, 2016). This approach is based on the hypothesis that knowledge of pharmacological targets and pathways together with toxicological and other data can provide sufficient information to anticipate the outcome of a 2-year rat carcinogenicity study and their potential value in predicting the risk of human carcinogenicity of a given pharmaceutical. The feasibility of this approach has been demonstrated in recent retrospective evaluations of carcinogenicity study results with pharmaceuticals (ICH, 2016, Sistare et al., 2011). A group of scientists from the pharmaceutical industry has published an analysis of a database consisting of 2-year carcinogenicity studies and chronic (6-months) toxicity studies in rats with 182 human pharmaceuticals (Sistare et al., 2011). The authors concluded that a lack of specific histopathological risk factors for neoplasia such as hyperplasia, foci of cellular alterations or chronic inflammation in a 26-week toxicity study in adult rats, together with lack of evidence for genotoxicity and for hormonal perturbation, can predict with 80% accuracy a negative outcome of the 2-year rat carcinogenicity study for these compounds. The approach is termed the “Negative for Endocrine, Genotoxicity, and Chronic study Associated histopathological Risk factors for Carcinogenicity in the Rat” (NEG CARC Rat) testing paradigm. Applying this prediction paradigm to new pharmaceuticals has the capacity to significantly reduce the number of 2-year rat carcinogenicity studies (40–50%), animals and costs without risk to human safety (Sistare et al., 2011). Using the same dataset complemented with study results from an additional 73 pharmaceuticals (255 compounds in total) the relationship of the carcinogenicity study outcome with the pharmacological properties of the compounds was analysed (van der Laan et al., 2016). The outcome of this analysis supports the notion that certain pharmacological drug classes are associated with tumour patterns in specific tissues in rats indicating that the pharmacological properties are a frequent key factor for the carcinogenic mode of action of many pharmaceuticals. The knowledge of the pharmacological pathways of a certain pharmaceutical can thus be used together with the NEG CARC approach to predict the outcome (either positive or negative) of a rat lifetime carcinogenicity study and may thus allow for a waiver of this study type.

Based on these evaluations the posted ICH RND (ICH, 2016) proposes that cancer risk of a new pharmaceutical can be predicted from data described above with sufficient certainty to be classified into one of three categories:

**Category 1:** highly likely to be tumorigenic in humans such that a product would be labelled accordingly and 2-year rat, 2-year mouse, or transgenic mouse carcinogenicity studies would not add value.

**Category 2:** the available sets of pharmacologic and toxicological data indicate that tumorigenic potential for humans is uncertain and rodent carcinogenicity studies are likely to add value to human risk assessment. Accordingly, current S1B Guidance (ICH S1B, 1997) describes options for rodent carcinogenicity testing.

**Category 3a:** highly likely to be tumorigenic in rats but not in humans through prior established and well recognized mechanisms known to be human irrelevant, such that a 2-year rat study would not add value; or

**Category 3b:** highly likely not to be tumorigenic in either rats or humans such that no 2-year rat study is needed. A study in a transgenic mouse could prove useful for justifying a Category 3 assignment.

For the categories 1, 3a and 3b a request for waiving the 2-year rat carcinogenicity study would be justified since the expected study results do not add any value to human risk assessment. In order to investigate the accuracy of this approach a prospective evaluation is currently in progress. Industry sponsors are encouraged to submit a Carcinogenicity Assessment Document (CAD) to DRAs in the ICH regions for all investigational pharmaceuticals with ongoing or planned 2-year rat carcinogenicity studies with a prediction of the carcinogenic potential prior to knowing the outcome of the carcinogenicity testing. A set of weight-of-evidence factors (Table 1) should be applied for predicting the outcome of the rat 2-year study and assignment to the above mentioned categories in the CAD. DRAs from each region will independently review the submitted assessments to evaluate the degree of concordance with sponsors and between regulatory regions. Predictions in the submitted CADs are then checked against the actual outcome of the 2-year rat studies as they are completed and reported to the DRAs.

**Table 1:** Weight-of-evidence factors for consideration in a Carcinogenicity Assessment Document (adapted from (ICH, 2016)).

Weight of evidence factors
1. Knowledge of intended drug target and pathway pharmacology, secondary pharmacology, & drug target distribution in rats and humans
2. Genetic toxicology results
3. Histopathologic evaluation of repeated dose rat toxicology studies
4. Exposure margins in chronic rat toxicology studies
5. Metabolic profile
6. Evidence of hormonal perturbation
7. Immune suppression
8. Special studies and endpoints
9. Results of non-rodent chronic study
10. Transgenic mouse carcinogenicity study

In March 2016 a status report provided an overview of the progress of the prospective evaluation study (ICH Status Report, 2016). From August 2013 to September 2015, the DRAs received and reviewed 25 CADs. Table 2 summarises the categories proposed by the sponsors and the corresponding categories chosen by the DRAs. The most remarkable differences between industry sponsors and regulators assignment was with regard to submitted category 3 cases. For 16 out of 25 (64%) cases the sponsors proposed category 3, split evenly between categories 3a and 3b and requested a (hypothetical) waiver. The DRAs concurred with only 6 of the category 3a/b cases and disagreed in 7 cases, concluding instead that these compounds should fall under category 2, and a 2-year study would have added value. For the remaining 3 category 3a/b cases proposed by sponsors, the DRAs could not reach consensus resulting in only partial alignment for category 3a/b. A diverging assignment occurred also with one case of a category 1 CAD (*i*.*e*., clear human risk, 2-year rat study not needed) submitted by a sponsor for which the DRAs concluded that a 2-year study could add value by defining the severity of the cancer risk, and therefore arrived at a category 2 designation (ICH Status Report, 2016). In the meantime (September 2016), additional 11 CADs and 7 reports of completed 2-year rat carcinogenicity studies have been submitted to the DRAs for review.

**Table 2:** Category designation by sponsors and Drug Regulatory Agencies for Carcinogenicity Assessment Documents (adapted from (ICH, 2016)).

Categories	Sponsor	DRAs
Category 1	2	1
Category 2	7	15
Category 3a	8	5
Category 3b	8	1
Partial DRA alignment	0	3

For a successful completion of the prospective evaluation study it has been agreed that a dataset of at least 20 cases for category 3 (*i*.*e*., CAD + study report) should be available which is expected to occur by the end of 2019 (ICH Status Report, 2016) (Table 2). Based on the degree of accuracy of predictions and of concordance among all parties conditions will be defined under which a weight-of-evidence evaluation to predict human cancer risk is an appropriate alternative to a 2-year rat carcinogenicity study. If this is the case then ICH guideline modifications involving waivers for the conduct of the 2-year rat study can be considered.

## 5 Integrating mechanistic information to support cancer hazard identification

Mechanistic data can provide evidence of carcinogenicity and can also help in interpreting the relevance and importance of findings of cancer in epidemiological and animal toxicology studies (International Agency for Research on Cancer, 2006). However, the volume and complexity of mechanistic data is increasing, comprising a diversity of human observational, experimental toxicology, as well as high-throughput and high-content data streams (Guyton et al., 2009, Straif et al., 2014). Another challenge is that agents can contribute to carcinogenesis in a number of different ways. Indeed, human carcinogens can operate through distinct mechanisms, with the genotoxic epoxide ethylene oxide and the AhR agonist 2,3,7,8-tetrachlorodibenzo-*para*-dioxin as notable examples. On the other hand, many human carcinogens (*e*.*g*., benzene, aflatoxin B1, asbestos) act *via* multiple mechanisms (Guyton et al., 2009). Moreover, there is no broadly accepted, systematic method for evaluating mechanistic data for the purpose of decision-making in cancer hazard identification. An important gap is the lack of a defined method or workflow to search systematically for data on relevant mechanisms.

An international Working Group of experts convened by the International Agency for Research on Cancer (IARC) identified 10 key characteristics, one or more of which are commonly exhibited by established human carcinogens (Smith et al., 2016). The ten characteristics are distinct from the hallmarks of cancer in reflecting carcinogen mechanisms. They represent the established properties of human carcinogens:

1. is electrophilic or can be metabolically activated;
2. is genotoxic;
3. alters DNA repair or cause genomic instability;
4. induces epigenetic alterations;
5. induces oxidative stress;
6. induces chronic inflammation;
7. is immunosuppressive;
8. modulates receptor-mediated effects;
9. causes immortalization; and
10. alters cell proliferation, cell death, or nutrient supply.

These characteristics provide the basis for an objective approach to identifying and evaluating evidence from pertinent mechanistic studies (Smith et al., 2016). The 10 key characteristics are used to systematically search the literature for evidence on relevant endpoints, and support objective evaluation of the overall strength of mechanistic information. Additionally, mapping of high-throughput assays to the 10 key characteristics can facilitate systematic evaluation as an additional mechanistic data stream.

Recent IARC Monograph evaluations demonstrate the applicability of this approach for mechanistically diverse agents (*e*.*g*. Grosse et al., 2016, International Agency for Research on Cancer, 2017, Loomis et al., 2015). For some compounds, there was strong evidence for only one (2,4-dichlorophenoxyacetic acid) or no (parathion) key characteristics (Guyton et al., 2015, Loomis et al., 2015). Interestingly, evidence for two key characteristics (is genotoxic, induces oxidative stress) was found for glyphosate, diazinon and malathion, with malathion additionally showing three others (induces chronic inflammation, modulates receptor-mediated effects, alters cell proliferation, cell death or nutrient supply) (Guyton et al., 2015). Dichlorodiphenyltrichloroethane (DDT) and tetrabromobisphenol A had strong evidence for a different set of key characteristics (modulates receptor-mediated effects, is immunosuppressive, and induces oxidative stress) (Grosse et al., 2016, Loomis et al., 2015).

High-throughput assay systems, such as the US EPA's ToxCast program (Kavlock et al., 2012, Tice et al., 2013), offer advantages of facilitating comparisons of activity across the hundreds of compounds evaluated by IARC, and can also support or fill gaps in evidence from *in vivo* experiments. Mapping of these assays to the key characteristics of carcinogens revealed substantial coverage for several, with support for traditional toxicology studies of “induces oxidative stress” and “alters cell proliferation, cell death or nutrient supply” and filling gaps for “modulates receptor-mediated effects” (Grosse et al., 2016, International Agency for Research on Cancer, 2017, Loomis et al., 2015). However, some key characteristics had little or no assay coverage in ToxCast, highlighting the need for additional high-throughput assays to provide more comprehensive coverage of the 10 key characteristics of carcinogens that form the basis of current IARC mechanistic evaluations. These developments lay groundwork for future evaluations where such data may fill important gaps in evidence of carcinogenicity.

## 6 Use of complementary mechanistic approaches to identify likely breast carcinogens

**Fig. 1:**
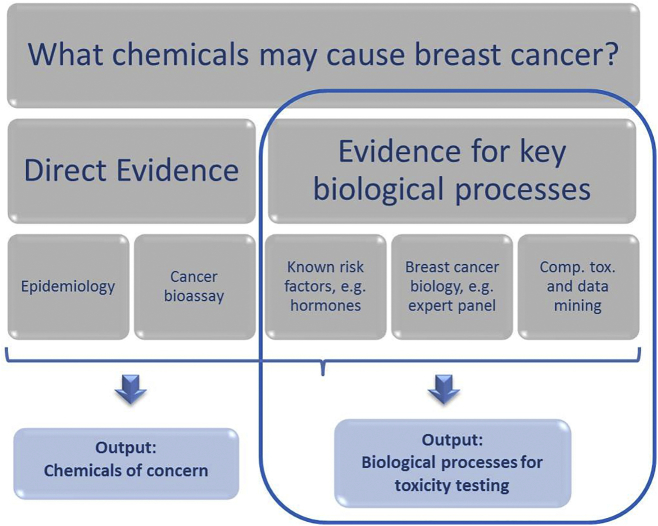
Identifying breast carcinogens using direct and indirect evidence. Outputs include chemicals of concern as well as biological processes to be included in toxicity screening. The steps in the blue box are the focus of the indirect evidences discussed within the text. (For interpretation of the references to color in this figure legend, the reader is referred to the web version of this article.)

Current approaches to chemical screening, prioritization, and assessment are being re-envisioned, driven by innovations in chemical safety testing, new regulations, and demand for information on human and environmental impacts of chemicals. One approach is to start with the disease reversing the usual direction of inquiry. It was chosen to begin with a disease outcome - breast cancer - to identify biological processes that play a role in etiology so that these processes could be the focus of chemical toxicity screening (Fig. 1). Three complementary approaches provide valuable insight into biological mechanisms for breast cancer.

First, known preventable risk factors for breast cancer include medical radiation, aspects of reproductive history, increased body weight for post-menopausal cancers, lack of physical exercise, alcohol consumption, pharmaceutical hormones, and probably tobacco smoke (IARC, 2012, IBCERCC, 2013, IOM, 2011). The mechanistic basis for many of these associations has been investigated and indicates biological processes relevant to breast cancer. Notably, several of these risk factors represent chemical exposures, suggesting that exposure to other chemicals with similar properties and biological activity (such as other hormonally-active chemicals, solvents or combustion products) may also pose preventable risks. Indeed, laboratory evidence suggests at least three overlapping classes of chemicals that increase breast cancer risk: 1) chemicals that cause mammary gland tumours primarily by damaging DNA, 2) endocrine-disrupting compounds that accelerate the growth of mammary tumours through estrogenic or other pathways, and 3) developmental toxicants that can alter development of the mammary gland in ways that permanently alter susceptibility (Brody and Rudel, 2008).

In a second approach, an expert panel of breast cancer biologists identified key biological processes whose perturbation may alter breast cancer risk, including cellular and molecular events, tissue changes, and factors that alter susceptibility (Table 3, from (Schwarzman et al., 2015)). A similar approach has been conducted to identify characteristics of carcinogens in the IARC process as described above (Smith et al., 2016) and biological activity important for carcinogenesis as part of the Halifax Project on Cancer (Goodson et al., 2015). The biological endpoints identified by a breast cancer expert panel were compared with those currently included in US ToxCast and Endocrine Disruptor Screening Program and identified important endpoints not currently evaluated by these testing programs, including altered mammary gland development, Her2 activation, progesterone receptor activity, prolactin effects, and aspects of estrogen receptor β activity.

**Table 3:** Biological Process Relevant to Breast Cancer Etiology (table from Schwarzman et al., 2015).

Cellular & molecular events	Tissue changes	Susceptibility factors
Actions in hormone levels, metabolism, or receptors Cell cycle changes Changes in transcription, translation, and epigenetic programming of genes associated with breast cancer Altered activity or expression of peptide hormones (growth hormones) Immune modulation Inflammation Oxidative stress Genotoxicity Limitless replication potential^a^ Evasion of apoptosis^a^ Autocrine growth^a^	Altered mammary gland development Terminal end bud proliferation Ductal hyperplasia Atypical hyperplasia Increased breast density/stromal hyperplasia Adenomas Carcinoma *in situ* Tissue invasion^a^ Sustained/enhanced angiogenesis^a^	Early onset of puberty Increased lifetime duration of estrogen exposure (early menarche or late menopause) Alterations in cyclicity Atypical function of metabolizing enzymes Obesity

a Indicators consistent with the hallmarks of cancer progression as defined by (Hanahan and Weinberg, 2011).

Third, computational toxicology approaches can be used to identify biological activities demonstrated by chemicals known to induce rodent mammary gland tumours or to alter mammary gland development. For example, lists of such chemicals have been compiled (Rudel et al., 2014, Rudel et al., 2007, Rudel et al., 2011) and their activity profiles in US EPA's ToxCast *in vitro* screens are compared to identify common mechanisms of action among these chemicals. This project will identify breast cancer-relevant mechanisms of action and also can highlight gaps in the biological space covered by ToxCast (Ackerman et al., in prep.).

Taken together, these complementary approaches identify mechanisms by which chemicals may alter breast cancer risk, so that testing for these mechanisms can be included in chemical screening batteries. This disease-focused approach to establishing chemical screening and testing methods can be extended to other cancers and disease outcomes, for example neurotoxicity, obesity, and diabetes (Auerbach et al., 2016, Thayer et al., 2012).

## 7 Integrated approaches for testing assessment (IATA)

Carcinogenicity testing has been recognized as the area with the most relevant needs for harmonisation among the different regulatory approaches. The standard approach usually starts with a battery of genotoxicity tests which is eventually followed up by a two-year rodent carcinogenicity bioassay (RCB) in case of positive results (Jacobs et al., 2016). With this conventional approach, non-genotoxic chemicals would be identified only when tested in the rodent cancer bioassay. Moreover, as stated above, the absence of genotoxic properties may provide the opportunity for waiving the cancer bioassay, with the risk that some non-genotoxic carcinogens remain unidentified.

The cell transformation assay (CTA) has been long debated as a possible *in vitro* test to study carcinogenesis. However, in 2014, the OECD reached the conclusion that it should not be used as a stand-alone assay to predict carcinogenesis in the regulatory context, but it could be useful in a weight-of-evidence approach, together with other test information. Due to the limitations of the current approaches to properly address the identification of non-genotoxic chemicals, the scientific and regulatory community recognized the need of developing an integrated approach to testing and assessment (IATA) for non-genotoxic carcinogenesis. An IATA is a science-based approach, which uses all the existing information integrated with new data. In this integrated approach, it is possible to consider the CTA as one of the possible building blocks of the IATA. All CTA models provide morphological endpoints of onco-transformation, which can be used as phenotypic anchoring for mechanistic studies. An experimental protocol which combined the BALB/c 3T3 CTA and a global gene expression analysis was developed to highlight the cross-talk between genotoxic and non-genotoxic carcinogenic mechanisms in the pathway leading to malignant cell transformation (Vaccari et al., 2015). Recent evidence suggests that the early steps in cancer initiation may be related to non-genotoxic carcinogenic mechanisms, which can drive genome instability and mutagenesis. In this integrated approach the reference chemical 3-methycholanthrene, a genotoxic chemical able to induce *in vitro* cell transformation, was used at both transforming and non-transforming concentrations in the BALB/c 3T3 CTA, to evaluate the gene modulation at critical steps of the experimental protocol. Results show that the initiating event is related to the activation of AhR signal transduction at both tested concentrations (Mascolo et al., in preparation). However, in the early step at the highest concentration, this activation is followed by the modulation of immune response and inflammation-related processes. These responses are distinctive signatures of the cells treated with the highest concentration, which acquire a fully malignant phenotype. Whereas, the lowest concentration induces activation of detoxifying pathways consequent to the activation of estradiol metabolic pathways.

The toxicogenomics approach applied to the *in vitro* CTA allowed the identification of the transcriptionally activated pathways, which are significantly modulated as the consequence of exposure by MCA and are strictly related to the formation of the transformed phenotype. This integrated approach has the potential to be considered to be part of an IATA for non-genotoxic carcinogenesis. Indeed, CTAs offer a good phenotypic anchoring, which links the cause (exposure) and the initial molecular key events to the event of onco-transformation.

## 8 Cross-Omic approaches

The *in vitro/in vivo* test battery for assessing genotoxicity and chemical carcinogenicity has been used for four decades now. Its practical usefulness for regulatory toxicology has abundantly been demonstrated, but as discussed above, several issues concerning its relevance for reliably predicting human cancer risks have emerged including low specificity despite high sensitivity and frequent overestimation of carcinogenic risks (Kirkland et al., 2005, Paules et al., 2011). Also, the current *in vitro* test strategy is not capable of capturing potential non-genotoxic carcinogens. The advent of the so-called “omics” technologies to toxicology, now more than a decade ago, has been widely held to provide the contingency for this. For instance, the EU's chemical policy program REACH considers the potential of these data-dense technologies for elucidating mechanisms-of-action which “permit assessment of a broad array of molecular changes that might be useful in the identification of potential carcinogens”. This concept has been applied to the field where a range of *in vitro* and *in vivo* studies at relatively large scale have been undertaken to generate whole genome gene expression profiles for classifying chemical carcinogens (for an overview of available studies and for accessing this data.(http://www.dixa-fp7.eu/ and Hendriks et al., 2016). Within this context, the outcome of the work under the EU FP7 project carcinoGENOMICS is of particular interest.

The carcinoGENOMICS project was set out to develop *in vitro* assays for predicting genotoxicity and carcinogenicity of the liver, kidney and lung, representing the main target organs in the rodent 2-year cancer bioassay. Per target organ, a comprehensive set of 30 test compounds, comprising genotoxic carcinogens, non-genotoxic carcinogens and non-carcinogens was selected (Vinken et al., 2008). For the lung, differentiated human bronchial epithelial cells in air liquid interface cultures appear to represent the most promising alternative (Boei et al., 2016). After evaluating multiple cell models the HepaRG cell line was selected to represent the liver (Doktorova et al., 2013) and the RPTEC/TERT1 immortalized renal tubular system as the preferred kidney model (Aschauer et al., 2013, Aschauer et al., 2015). When subjected to challenges by the test compounds, the HepaRG (Doktorova et al., 2014) and the RPTEC/TERT models appeared to achieve an approximately 85% level of accuracy in correctly identifying carcinogen classes. In addition, both systems were assessed for inter-laboratory variability and resulted to be quite robust (Herwig et al., 2016). Independently, a predictive transcriptomics-based model was developed for assessing genotoxicity *in vivo* using the HepG2 cell line and evaluating a set of about 60 prototypical carcinogens. This resulted in prediction of accuracies that were even higher when combining gene expression profiles with results from the classical Ames mutagenicity test (Magkoufopoulou et al., 2012).

These and other studies demonstrated that gene expression patterns consisting of several tenths of genes can be developed which may outperform the existing *in vitro/in vivo* test battery for predicting genotoxicity/carcinogenicity.

MicroRNAs are small non-coding RNA sequences capable of silencing mRNAs thereby down-regulating gene expression. A single microRNA may target multiple different mRNAs. Hypothetically, predictive profiles may thus be generated which consist of only a few microRNAs. This was investigated by retrieving global microRNA data from primary mouse hepatocytes treated with 21 prototypical carcinogens. However, no microRNA profile capable of neither discriminating genotoxins from non-genotoxins, nor non-genotoxic carcinogens from non-carcinogens could be identified. Samples from the same experiments were also subjected to whole genome gene expression analysis, and again, accurate classifying transcriptome patterns could be identified (Rieswijk et al., 2016a).

Other large-scale attempts of creating cross-omics profiles for classifying genotoxins/carcinogens, by exploring the proteome or the epigenome, have not yet been reported. By contrast, cross-omics analysis, in particular when involving also the investigation of epigenomic changes, may considerably deepen our understanding of carcinogenic mechanisms of action. In order to evaluate whether the epigenome is subject to change at all when exposed *in vitro* to carcinogens, an analysis of DNA methylation patterns was undertaken in A549 human adenocarcinoma lung cells exposed to arsenic for up to 14 days. Dose- and time-dependent effects on the methylome, paralleled to some degree with concordant gene expression modulations known to play a role in lung cancer promotion and progression. In particular, the induction of a tumour protein linked to a p53-centered network was observed (van Breda et al., 2015).

Based on these promising results, the analysis of epigenome-transcriptome-microRNA interactions was initiated in primary human hepatocytes pooled from 3 donors to bypass confounding factors due to inter-individual variability in susceptibility, and exposed for 5 days to the human carcinogen aflatoxin B1 (AFB1). Substantial AFB1-induced changes in DNA methylation patterns affecting 5000–6000 genes were observed, while the transcriptome responses involved 1500–2500 differentially expressed genes. About 20 differentially expressed microRNAs were identified only at relatively high AFB1 dose. For the purpose of data reduction and, in addition, for translating responses observed *in vitro* to the human disease state, a gene expression signature was derived from samples of hepatocellular carcinoma patients (reported in literature). Data on gene expression and DNA methylation levels of overlapping genes were fed into a pathway finding resource, and complemented with data on differentially expressed microRNAs. Through this approach, *a priori* knowledge on gene-gene interactions induced by AFB1 treatment was confirmed. Moreover, numerous new interactions where identified, which refer mainly to development- and cell differentiation-related processes. Interestingly, a large part of the AFB1-induced methylome changes appeared persistent for 3 days upon terminating AFB1 exposure (Rieswijk et al., 2016b).

It appears that transcriptome profiling *in vitro* brings added value for predicting genotoxicity/carcinogenicity *in vivo* and a cross-omics approach may not even be required for improving accuracy of prediction. Interestingly, over the last years substantial transcriptomic data sets on genotoxicants and carcinogens have become available (Hendriks et al., 2016), which should be further explored for this purpose. Cross-omics investigations seem very promising for hypothesis generation and retrieving in depth insights on novel mechanisms-of-action, thus substantiating the development of relevant Adverse Outcome Pathways.

## 9 Conclusions

The paradigm shift in carcinogenicity assessment, which is driven by progress in chemical safety testing and increasing demand for information on human and environmental impact of chemicals, applies to all categories of substances. As emerged by the contributions to the workshop, the development of novel approaches to ensure an accurate carcinogenicity hazard assessment is needed for substances where cancer bioassay in rodents is still a basic requirement, as well as for those substances where animal use is banned or limited or where information gaps are identified within legislations. The introduction of a new generation of products into the market, such as biopesticides and biomedicines, is creating fresh regulatory challenges for adequate safety evaluation which requires new tools. Non-genotoxic carcinogens are also of particular concern. Under many chemical regulations the cancer bioassay is rarely required, specific requests to obtain information on non-genotoxic mechanisms of carcinogenicity are few, and there are no OECD approved screening methods. Therefore, these substances may remain undetected and the risk they may pose to human health may not be managed adequately (Jacobs et al., 2016).

**Fig. 2:**
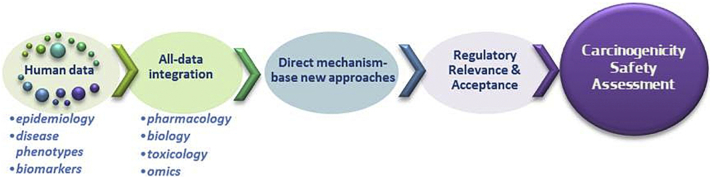
Moving forward in carcinogenicity assessment. Integration of data, application of new technologies and approaches, regulatory relevance and acceptance across sectors can warrant a better carcinogenicity safety assessment.

It is evident that a number of initiatives utilising newer approaches are emerging, including data-driven or knowledge-driven approaches and those that focus on a disease outcome to establish chemical screening and testing methods. However, there is a need to direct efforts towards the integration of all available information on relevant endpoints, including from epidemiology, traditional and alternative toxicology test systems, and from novel data streams (Fig. 2). This is confounded by a significant disconnection between current regulatory frameworks and the practical application of already existing novel approaches and mechanistic understanding. For the purpose of regulatory decision-making in cancer hazard identification a systematic method for evaluating mechanistic data has been implemented by IARC and a structured way to integrate this type of information in an IATA is currently being investigated at international level by the OECD in relation to non-genotoxic carcinogenicity. This may help in bridging the gap between legal requirements and scientific needs. In this context it will be essential to take stock of the performance of the actual methods in order to define a benchmark that new approaches should overcome. Moreover, the generation of new data, utilising omics-based assays, together with traditional carcinogenicity endpoints in human non-cancerous cell culture models, will facilitate acquiring the knowledge required to drive hypothesis formulation and to create a new set of testing criteria before more far-reaching solutions emerge which can stand up to the demands of regulatory testing.

For specific product areas waiving opportunities as well as the use of mode of action information to support informed decisions on human relevance are already being considered at regulatory level. However, in order to fully exploit these approaches and the increased molecular understanding of carcinogenicity, it is critical to share experiences and approaches and to explore their applicability across all sectors. Such cross-sectorial harmonisation will aid in building momentum for new approach method implementation and will reduce the reliance on the two-year rodent bioassay.

## Transparency document

Transparency document Image 1
